# Histopathologic Composition of Cerebral Thrombi of Acute Stroke Patients Is Correlated with Stroke Subtype and Thrombus Attenuation

**DOI:** 10.1371/journal.pone.0088882

**Published:** 2014-02-11

**Authors:** Joris M. Niesten, Irene C. van der Schaaf, Lievay van Dam, Aryan Vink, Jan Albert Vos, Wouter J. Schonewille, Peter C. de Bruin, Willem P. T. M. Mali, Birgitta K. Velthuis

**Affiliations:** 1 Department of Radiology, University Medical Center Utrecht, Utrecht, The Netherlands; 2 Department of Pathology, University Medical Center Utrecht, Utrecht, The Netherlands; 3 Department of Radiology, St. Antonius Hospital, Nieuwegein, The Netherlands; 4 Department of Neurology, St. Antonius Hospital, Nieuwegein, The Netherlands; 5 Department of Pathology, St. Antonius Hospital, Nieuwegein, The Netherlands; INSERM U894, Centre de Psychiatrie et Neurosciences, Hopital Sainte-Anne and Université Paris 5, France

## Abstract

**Introduction:**

We related composition of cerebral thrombi to stroke subtype and attenuation on non-contrast CT (NCCT) to gain more insight in etiopathogenesis and to validate thrombus attenuation as a new imaging biomarker for acute stroke.

**Methods:**

We histopathologically investigated 22 thrombi retrieved after mechanical thrombectomy in acute stroke patients. First, thrombi were classified as fresh, lytic or organized. Second, percentages of red blood cells (RBCs), platelets and fibrin and number of red, white (respectively RBCs or platelets outnumbering other components with ≥15%) or mixed thrombi were compared between large artery atherosclerosis (LAA), cardioembolism, dissection and unknown subtype. Third, correlation between attenuation and RBCs, platelets and fibrin was calculated using Pearson's correlation coefficients (*r*).

**Results:**

Thrombi were fresh in 73% (n = 16), lytic in 18% (n = 4) and organized in 9% (n = 2). The stroke cause was LAA in eight (36%), cardioembolism in six (27%), dissection in three (14%), and unknown in five (23%) patients. LAA thrombi showed the highest percentage RBCs (median 50 (range 35–90)), followed by dissection (35 (20–40), p = 0.05), cardioembolism (35 (5–45), p = 0.013) and unknown subtype (25 (2–40), p = 0.006). No differences in platelets (p = 0.16) and fibrin (p = 0.52) between subtypes were found. LAA thrombi were classified as red or mixed (both n = 4), cardioembolisms as mixed (n = 5) or white (n = 1) and dissection as mixed (n = 3). There was a moderate positive correlation between attenuation and RBCs (r = 0.401, p = 0.049), and weak negative correlations with platelets (r = −0.368, p = 0.09) and fibrin (r = −0.073, p = 0.75).

**Conclusions:**

The majority of cerebral thrombi is fresh. There are no differences in age of thrombi between subtypes. LAA thrombi have highest percentages RBCs, cardioembolism and unknown subtype lowest. No relationship exists between subtype and platelets or fibrin percentages. We found a correlation between the RBC-component and thrombus attenuation, which improves validation of thrombus attenuation on NCCT as an imaging biomarker for stroke management.

## Introduction

Thromboembolism is the cause of ischemic stroke in the majority of acute stroke patients [1]. These thrombi originate mainly from large artery atherosclerosis (LAA) and cardioembolisms, and more infrequently from dissection [2]–[4].

The use of mechanical thrombectomy devices allows retrieval of cerebral thrombi from the intracranial vessels and subsequent histopathologic analysis. The first histopathologic studies showed that the architecture of cerebral thrombi is variable with different main components such as fibrin, red blood cells (RBC) and platelets [5]–[7]. The composition of thrombi may influence the efficacy of thrombolysis with recombinant tissue plasminogen activator (rtPA) since RBC-rich or red thrombi are described to be more vulnerable for current thrombolysis compared to platelet-rich or white thrombi [8]–[10].

Furthermore, the age of thrombi could also play a role in success rate of thrombolysis, which is only about 50% with current IV-rtPA therapy. Recanalisation depends on the time from stroke onset to intravenous (IV) rtPA-treatment, hence it may be more effective in fresh than old thrombi [11].

On non-contrast CT (NCCT) platelet-rich thrombi have lower attenuation than RBC-rich thrombi since the attenuation has a linear correlation with the concentration of hemoglobin [9], [12]–[14]. Recent studies found that the attenuation is also related to stroke subtype [15], [16] and could be helpful in predicting the rate of recanalisation after rtPA [17], [18].

Improved knowledge about thrombus age and composition and its relation with stroke subtypes and attenuation could help to gain more insight in the etiopathogenesis of stroke. The aims of this study were to relate the age and histopathologic composition of thrombi to the stroke subtype and to thrombus attenuation on NCCT.

## Methods

### Patient population

We histopathologically investigated thrombi retrieved after mechanical thrombectomy of acute stroke patients admitted to the University Medical Center Utrecht or the St. Antonius Hospital Nieuwegein, between December 2010 and April 2013. Inclusion criteria were: age ≥18 years, National Institutes of Health Stroke Scale (NIHSS) ≥2, acute stroke patients with an intracranial thrombus on CT-Angiography (CTA) eligible for thrombectomy, retrieval of thrombus material available for histopathologic analysis. Exclusion criteria were the collection of thrombus material that was unsuitable for histopathologic analyses and a poor quality NCCT that was unsuitable for thrombus density measurements.

The following baseline data were collected: demographic features, cardiovascular risk factors (hypertension, diabetes mellitus, smoking), NIHSS on admission, location of the thrombus, and onset time to CT-imaging, to IV-rtPA treatment and thrombus retrieval.

LAA was defined as imaging findings of either significant (>50%) stenosis or occlusion of a major cervical (carotid or vertebral) artery or intracranial artery on CTA ipsilateral to the symptomatic hemisphere, presumed to be due to atherosclerosis according to the Trial of Org 10172 in Acute Stroke Treatment (TOAST) criteria [19] if other diagnostic studies had excluded potential sources of cardiac embolism. Cardioembolism was defined as at least one cardiac source for an embolus identified in the absence of significant ipsilateral stenosis (>50%) of ipsilateral large extracrianal arteries or atherosclerosis. Criteria for diagnosing dissection as the cause of stroke were a narrowed eccentric lumen surrounded by crescent-shaped mural thickening or tapered occlusion with associated increase in external vessel diameter on CTA [20], [21].

### Ethics statement

The medical ethics committee of the University Medical Center Utrecht approved the study. If patients had the capacity to consent, written informed consent was obtained from themselves. If this was not the case, written informed consent was obtained from their nearest relative(s).

### Treatment prior to thrombectomy

Based on clinical and imaging information IV-rtPA was administered to patients when considered eligible by a team of experienced neuroradiologists and neurologists. If IV-rtPA failed or could not be administrated due to contra-indications, the administration of intra-arterial (IA) rtPA followed by thrombectomy or thrombectomy alone was done, in all cases under systemic heparin. The heparin was given IV at a dose of 2500 IU.

### Thrombectomy Procedure

Informed consent was obtained prior to the procedure. Mechanical thrombectomy was performed in accordance with local guidelines. All patients had heparin administered during the percutaneous intervention. The occlusion was reached using a guiding catheter through the femoral artery. The thrombus was removed by a Merci retriever, a Trevo retriever or a Solitair stent and thrombus material was collected in a formalin equipped bottle.

### Histopathologic Procedure

Specimens were embedded in paraffin and 4 consecutive 5-µm thick slices were cut. All thrombi were stained with hamatoxylin and eosin, Mallory's phosphotungstic acid-hematoxilin (identifying fibrin), glycophorin A (identifying RBCs) and CD31 (identifying platelets) immunostains.

Subsequently, thrombi were histopathologic analysed by an experienced vascular pathologist (A.V.) together with two other investigators (J.N. and L.v.D.) on all 4 slices. Histological analysis was conducted in a blinded fashion without any knowledge of patient and stroke characteristics. First, all thrombi were classified as fresh (<1 day), lytic (1–5 days) or organized (>5 days), based on previously published definitions of thrombus age [22]. Thrombi were classified according to the age of the largest part of the thrombus. Second, percentages of RBCs, platelets and fibrin were quantitatively determined in consensus. Subsequently, thrombi were classified as red if RBCs outnumbered platelets and fibrin by at least 15% and as white if platelets outnumbered RBCs and fibrin with at least 15% difference. If this was not the case, thrombi were classified as mixed. Furthermore, we also investigated the presence of other components (white blood cells, atheromatous material, calcification) in the thrombi.

### Scan protocol and attenuation measurements

All imaging studies were performed using 64- or 256-slice MDCT–scanner (Philips, Philips Healthcare, Best, the Netherlands). CT parameters of the NCCT were: 120 kVp, 300 mAs, and 3-mm reconstructed slice thickness. For the CTA 65–70 ml of contrast agent (300 mg Iopromide/mL) was injected into the antecubital vein (18-gauge needle) at a rate of 6 mL/s followed by a 40-mL saline flush at the same rate.

The attenuation of the thrombus was measured on 3-mm NCCT in Hounsfield Units (HU) by two independent observers, both blinded for any data except localization of the thrombus on CTA. Within the thrombus a standardised small round region of interest was drawn three separate times and mean HU-values were used for analysis. The same measurements were performed in the corresponding contralateral vessel and by calculating the relative HU (HU thrombus/HU contralateral) we corrected for hematocrit. If the thrombus was situated in the basilar artery, a more proximal non-thrombosed portion of the basilar artery was used for the control measurements. If the proximal end was not open, the supraclinoid ICA was used (n = 3).

### Statistical Analyses

Baseline characteristics were summarised as means (with standard deviation (SD)), medians (with range) or frequency counts and proportions.

First, the percentages of RBCs, platelets and fibrin were compared between the subtypes of stroke by the Kruskal-Wallis test. Significant group differences were further analysed by the Mann-Whitney U test to determine individual differences.

Intraclass correlation coefficients (ICCs) were used to evaluate the interobserver variation in HU-measurements of the thrombus. We considered ICC values <0.20 as poor, 0.21–0.40 as fair, 0.41–0.60 as moderate, 0.61–0.80 as good, and 0.81–1.00 as excellent. The correlation between attenuation and percentages of RBCs, platelets and fibrin was calculated using Pearson's correlation with a coefficient (*r*) of 0.90–1 as very strong; 0.70–0.89 as strong; 0.40–0.69 as moderate; 0.20–0.39 as weak and 0.01–0.19 as a negligible relationship. Statistical analysis was performed using SPSS (version 20.0, SPSS Inc., Chicago). Results were considered statistically significant if P-values were <0.05.

## Results

A total of 22 thrombi were retrieved from 22 patients with an average age of 60 years (±13) and 50% female *(* *Table 1* *)*. No patients had to be excluded. The median time from onset to thrombus retrieval of was 287 minutes (125–455). The mean NIHSS was 14, with a minimum of 5 and a maximum of 35. In 17 patients (77%) IV-rtPA was administrated with a median time of 88 minutes from onset. Only 2 patients (9%) were on chronic anticoagulation medication and 3 patients (14%) received IA-rtPA prior to mechanical thrombectomy. The cause of stroke was classified as LAA in eight (36%), cardioembolism in six (27%), dissection in three (14%), and as unknown in five (23%) patients.

**Table 1:** Patient characteristics.

Age in years, mean (±SD)	60 (±13)
Sex (female), n (%)	11 (50)
**Subtype, n (%)**	
Large artery atherosclerosis	8 (36)
Cardioembolism	6 (27)
Dissection	3 (14)
Unknown	5 (23)
**Cardiovascular risk factors, n (%)**	
Current smoking	10 (45)
Hypertension	9 (41)
Diabetes Mellitus	1 (5)
Chronic anti-coagulation use, n (%)	2, (9)
**Baseline NIHSS, median (range)**	14 (5–35)
**Intravenous thrombolysis (rtPA), n (%)**	17 (77)
**Intra-arterial thrombolysis (rtPA), n (%)_**	3 (14)
**Location thrombus, n (%)**	
Middle Cerebral Artery	14 (64)
Basilar Artery	7 (32)
Internal Carotid Artery	1 (5)
**Onset time to CT in minutes, median (range)**	107 (20–386)
**Onset time to retrieval in minutes, median (range)**	287 (125–455)
**Onset time to IV-rtPA in minutes, median (range)** *	88 (35–290)

* based on 17 patients received IV-rtPA

SD: standard deviation, NIHSS: National Institutes of Health Stroke Scale, rtPA: recombinant tissue plasminogen activator, CT: Computed Tomography

Thrombi were fresh in 73% (n = 16), as lytic in 18% (n = 4) and as organized in 9% (n = 2). No significant differences were found in the age of thrombi between the different subtypes (p = 0.54). All lytic and organized thrombi also revealed a component of the thrombus that was fresh. All thrombi that were fresh did not have any organized parts and only a very few fresh thrombi showed minimal lytic parts.

**Figure 1:**
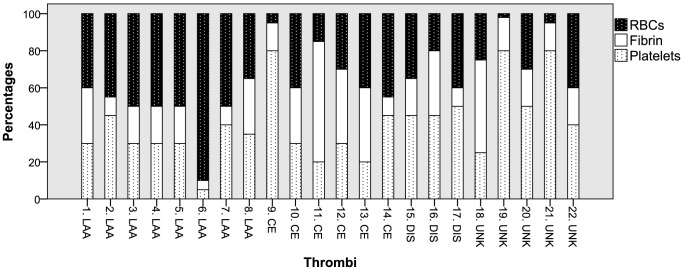
Histopathologic composition of all thrombi. RBC: Red blood cell.

The median (range) percentages of components across all thrombi were 40% (2–90%) RBCs, 40% (5–80%) platelets, and 20% (5–65%) fibrin *(* *Figure 1* *)*.

**Figure 2:**
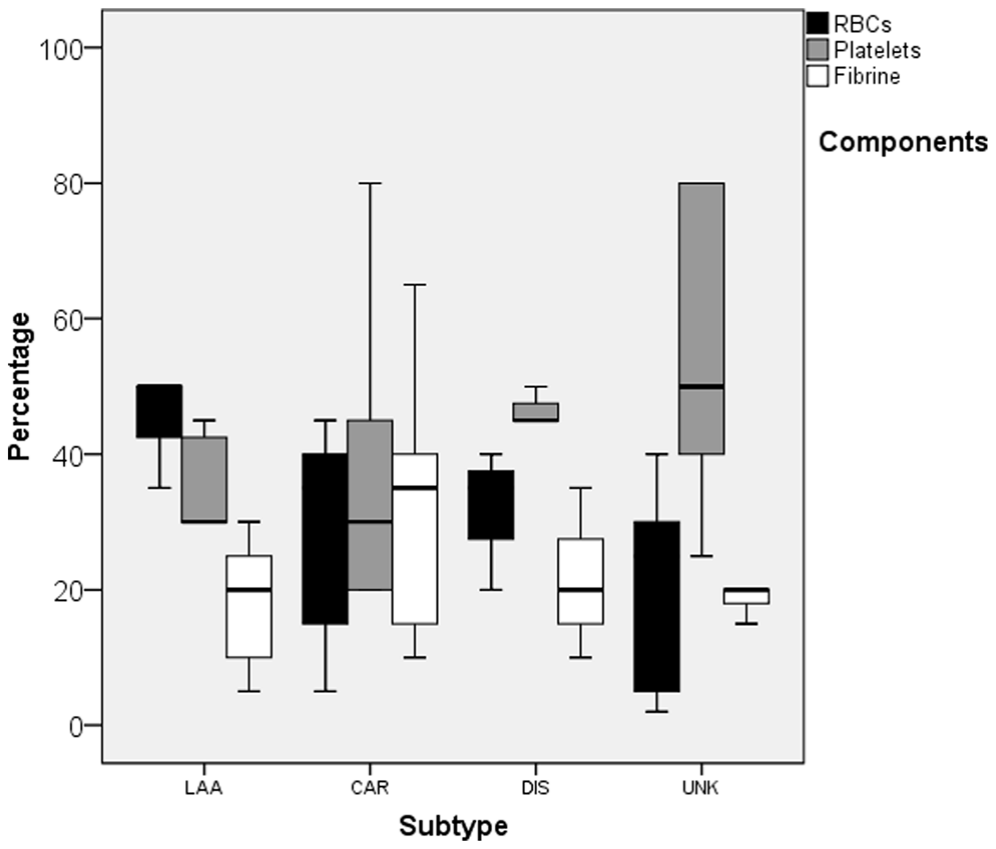
Boxplots of percentage red blood cells, platelets and fibrin of thrombi by subtype of stroke. LAA: large artery atherosclerosis, CE: cardioembolism, DIS: dissection, UNK: unknown, RBC: Red blood cell.

The median percentages of RBCs differed significantly between the different stroke subtypes as a group (p = 0.010) while there were no significant differences in the median percentages of platelets (p = 0.16) and fibrin (p = 0.52) between the different subtypes *(* *table 2* *and* *figure 2* *)*. Comparing the individual stroke subtypes; thrombi originating from LAA showed the highest percentage of RBCs (median 50% (35–90%)), which was not significantly different from the dissection subtype (median 35% (20–40%), p = 0.05) but was significantly higher than thrombi from cardioembolisms (median 35% (5–45%), p = 0.013) and unknown subtype (median 25% (2–40%), p = 0.006). The median percentages of RBCs in thrombi from dissection and cardioembolisms (p = 0.82), dissection and unknown subtype (p = 0.33) and cardioembolism and unknown (p = 0.39) were comparable *(* *Figure 2* *)*. White blood cells were present in most thrombi but in very small amounts only, in one thrombus a small volume of atheromatous material was seen and calcification was not visible in any thrombus.

**Table 2:** Thrombus components: relation with stroke subtypes and correlation with attenuation on non-contrast CT.

Component	Stroke subtype						Correlation with attenuation	
		*LAA*	*CE*	*DIS*	*UNK*	*P-value*		
*RBC*	*mean (range)*	50% (35–90%)	35% (5–45%)	35% (20–40%)	25% (2–40%)	0.010	*r (p-value)*	0.401 (0.049)
*Platelets*	*mean (range)*	30% (5–45%)	30% (20–80%)	45% (45–50%)	50% (25–80%)	0.16	*r (p-value)*	−0.368 (0.09)
*Fibrin*	*mean (range)*	20% (5–30%)	35% (10–65%)	20% (10–35%)	20% (15–50)	0.52	*r (p-value)*	−0.073 (0.75)

LAA: large artery atherosclerosis, CE: cardioembolism, DIS: dissection, UNK: unknown, RBC: Red blood cell.

**Figure 3:**
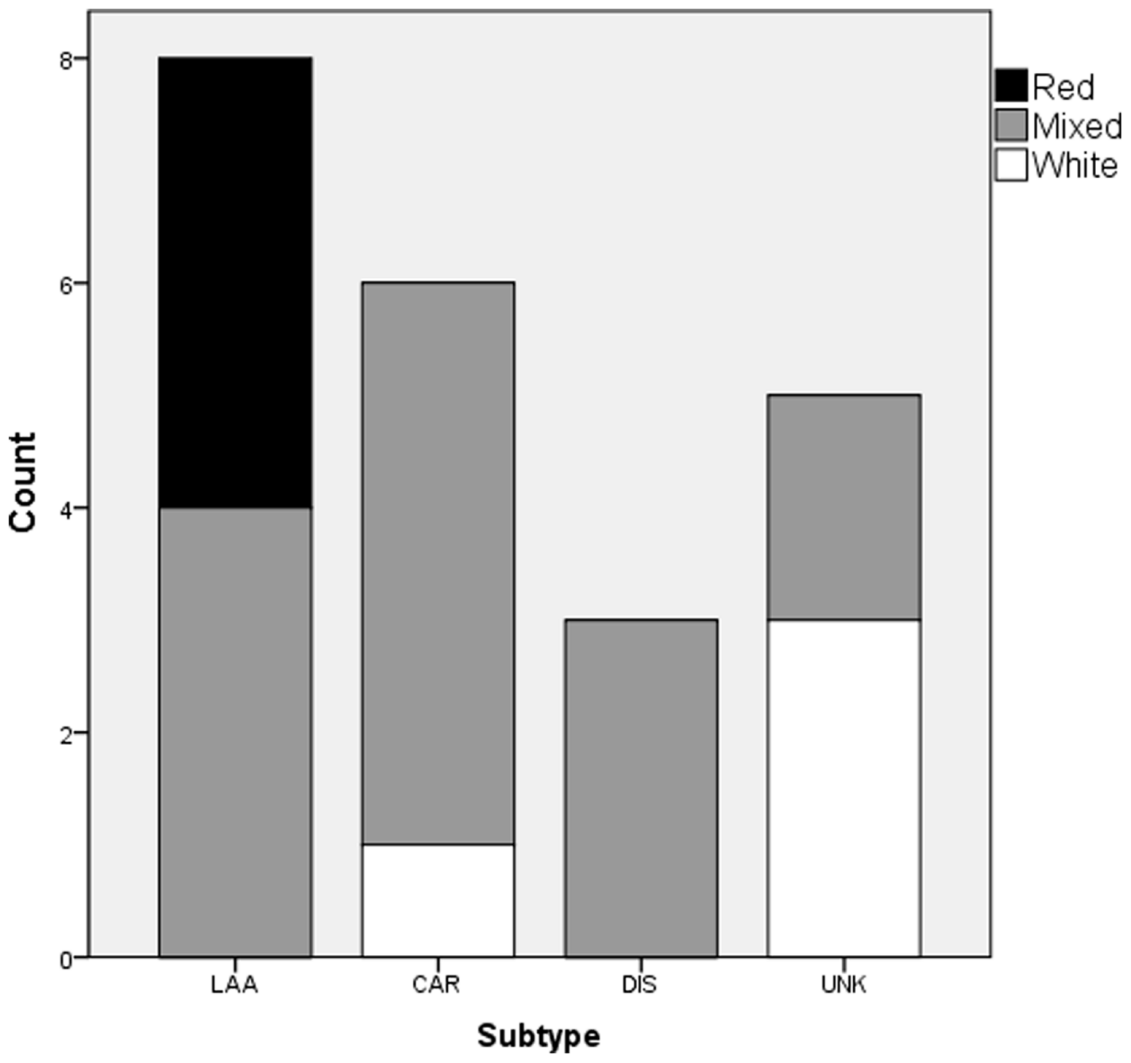
The number of red, white and mixed thrombi by subtypes. LAA: large artery atherosclerosis, CAR: cardioembolism, DIS: dissection, UNK: unknown.

Thrombi were defined as red (RBC-rich) in 4 (18%), as white (platelet-rich) in 4 (18%) and as mixed in 14 (64%) cases *(* *figure 3* *)*. Thrombi from LAA were classified as red in half of the cases (n = 4) and as mixed in the other half. Cardioembolic thrombi were defined 5 times (83%) as mixed and once as white (17%). All 3 dissection thrombi were classified as mixed, while the unknown subtype contained 3 white (60%) and 2 mixed thrombi (40%). No white thrombi were found in LAA and none of the cardioembolic, dissection, or unknown subtype thrombi were classified as red. Thrombi in patients with LAA had a significantly higher proportion of red thrombi than in cardioembolisms (p = 0.04), and significantly less white thrombi than the unknown subtype (p = 0.012) while differences between all other subtypes in number of red and white thrombi were insignificant (p>0.06).

The interobserver reliability for the HU-measurements was excellent (ICC = 0.98). There was a moderate linear correlation between the attenuation and percentage of RBCs (r = 0.401, p = 0.049), a weak negative linear correlation between attenuation and platelets (r = −0.368, p = 0.09) and a negligible negative linear correlation for fibrin (r = −0.073 p = 0.75, *table 2*).

## Discussion

We found that most cerebral thrombi were fresh and that thrombi originating from LAA had the highest percentage of RBCs and were the only stroke subtype with red thrombi. There was a moderate correlation between the percentage of RBCs and the attenuation on NCCT.

Traditional teaching emphasizes that fresh red thrombi, containing mixtures of fibrin and RBCs, originate from low flow-regions while white thrombi, existing mainly out of platelets and fibrin, arise in regions of fast moving blood [23], [24]. In cerebral stroke, thrombi from LAA are thought to originate from regions of fast moving blood, while most cardioembolic thrombi are assumed to develop from low flow-regions, such as the left atrial appendage. However, histopathology examination of cerebral stroke thrombi is limited and interpretations about the pathophysiology of cerebral thrombi have mainly been derived from coronary circulation studies.

Thrombi causing myocardial infarction originate from a local coronary artery occlusion due to the atherosclerotic process. In this process RBCs were formerly described to be of little importance [25]. However, more recently the influence of RBCs in coronary atherosclerosis has been described as major, especially in the transition from a stable to an unstable lesion causing occlusions [26]–[29]. It is therefore more likely that thrombi originating from (unstable) atherosclerotic plaques have a high RBC-component, especially in cerebral thrombi where the majority of atherosclerotic thrombi are due to acute thromboembolic events immediately after intraplaque hemorrhage [30]–[32].

In regard to the composition of cardioembolic thrombi, only one study investigated the histopathology of in vivo derived cardioembolic thrombi from patients with atrial fibrillation by either extraction from atrial thrombi during cardiac valve surgery (11 patients) or by removal of embolized cardioembolic thrombi from ilio-femoral and subclavian-brachial arteries during vascular surgery (11 patients) [33]. They found that all cardiac thrombi consisted mainly out of fibrin, platelets and debris. Furthermore, an important difference between cardiac in situ thrombus and embolized thrombi was found; embolized thrombi showed twice as much platelet-rich domains (40% of total) compared to non-embolized atrial thrombus material (20%).

Our findings are not in line with the traditional assumption. We observed that thrombus from LAA contain the highest amount of RBCs and that all red thrombi originate from LAA and none from cardioembolisms.

Our findings are, however, to a great extent in line with the previous histopathologic studies investigating cerebral thrombi, which also found that the traditional assumption is not valid for stroke patients. To our knowledge only three histopathologic studies were performed on thrombi retrieved from cerebral arteries of patients having acute stroke [5]–[7]. The first two studies, performing histological analyses of thrombi retrieved from intracranial vessels in respectively 5 and 25 patients with ischemic stroke, showed no relation between thrombus composition and stroke subtype [6], [7]. These investigators found similar cell components of thrombi derived from either arterial or cardiac sources and also demonstrated that atherosclerotic thrombi contain significant amounts of RBCs. The most recent histopathologic study measured the percentages RBCs, white blood cells and fibrin in 50 patients and related thrombus composition to stroke subtype [5]. They found a broad histopathologic distribution of thrombus composition, although white blood cells were consistently marginal and platelets were not investigated. They also mentioned that no relation between thrombus histopathology and stroke subtype was found. However, any other details about this relationship were not provided. In our study results we do not report percentages of white blood cells or other thrombus components because they were not or only in small amounts present.

Other than only one brief report on a thrombus from a single patient with a dissection, in which the histological examination was described as “serpentine” (fibrin:platelet bands interspersed with accumulations of nucleated cells and RBC-rich accumulations) [6], there are no studies available about the histology of strokes caused by dissection.

To our knowledge this is the first study investigating the age of thrombi in acute cerebral stroke patients. While the traditional assumption emphasizes that fresh thrombi originate mainly from cardioembolisms, we found no differences in age of thrombi between the stroke subtypes. Furthermore, we found a higher percentage of fresh thrombi compared to studies of myocardial infarction patients, in which at least half of the cardiac thrombi were days or weeks old [22]. This discrepancy increases the thought that the pathogenesis of cardiac and cerebral thrombi may be different. We also found that all lytic and organised thrombi also contained a component that was fresh (less than one day old). Fresh thrombus may occur around the local plaque rupture or an organised thrombus can embolize to the brain, incompletely occlude a brain artery, and then acquire fresh thrombus elements which may ultimately lead to complete vessel thrombosis.

As mentioned, thrombus attenuation could be related to the constituents and composition of the thrombus. The concentration of hemoglobin is one of the determining factors as it has a linear correlation with the attenuation while platelets and atheromatous or cellular debris are known to decrease the attenuation of the thrombus on CT [9], [12]–[14], [34]–[36]. Studies on this topic, however, are experimental, animal studies, or investigations of the venous sinus thrombus. Other than a recent histopathological study briefly mentioning that they did not find a significant relation between attenuation and composition [5], little is know about the relation between the attenuation of thrombi on NCCT and the histological composition of retrieved cerebral thrombi. We are the first study describing a correlation between the histopathological RBC-component of thrombi and the attenuation on NCCT. This correlation could be stronger in reality as attenuation was measured before IV-rtPA administration while most thrombi were histopathologically investigated after thrombolysis which could have influenced thrombus composition. As RBCs are more vulnerable for rtPA compared to platelets it is likely that the especially RBCs were influenced before histopathological examination.

There are some aspects that merit consideration. First, only a relative small number of thrombi were investigated, consequently making it hard to draw strong conclusions. Second, no clear definition of “red” and “white” thrombi exists which is probably due to the fact that just a few studies have pathologically assessed cerebral thrombi in vivo. Most histopathologic studies based the division in red or white thrombi on “macroscopic evaluation” without any thresholds [5], [6], [37]–[40]. To increase the objectivity and reproducibility, we introduced a cut-off value to distinguish between red and white thrombi. Third, the majority of the histopathologically investigated thrombi were resistant to IV-rtPA and we could not investigate thrombi with successful recanalization on IV-rtPA, This may bias the percentage of thrombus composition but not the correlation between histopathology and thrombus attenuation. Fourth, besides the administration of IV-rtPA, the mechanism and timing of the procedures also varied and could have influenced the thrombus composition. Therefore we cannot assess the predictive value of thrombus composition, but this was not the purpose of the study. Fifth, attenuation measurement were performed on 3-mm slices, the thinnest slices available for evaluation. Partial volume effects may have influenced our attenuation results since some thrombi could be smaller than 3 mm or not completely included in one slice. However, this would have probably only led to less differences between subtypes and thus resulting in an underestimation of the results.Due to these considerations larger histopathologic studies of prospectively collected cerebral thrombi are needed to validate our findings. Ideally, the attenuation measurements will be performed on thin slices and the predictive value of clot composition for recanalization after treatment will be determined.

## Conclusions

In our study with relatively small numbers important findings were seen: we found that the majority of cerebral thrombi is fresh and that there are no differences in age of thrombi between the stroke subtypes. Thrombi originating from large artery atherosclerosis have the highest percentages of red blood cells followed by thrombi due to dissection, while cardioembolic and unknown stroke subtype thrombi have the least red blood cells. A moderate correlation was found between the red blood cell-component and attenuation on non-contrast CT, improving the validation of thrombus attenuation on non-contrast CT as an imaging biomarker for acute ischemic stroke management. These findings will have to be validated in a larger study.
